# Blood Free-Circulating DNA Testing of Methylated RUNX3 Is Useful for Diagnosing Early Gastric Cancer

**DOI:** 10.3390/cancers12040789

**Published:** 2020-03-26

**Authors:** Eizaburou Hideura, Yutaka Suehiro, Jun Nishikawa, Takuya Shuto, Hiroyuki Fujimura, Shunsuke Ito, Atsushi Goto, Kouichi Hamabe, Issei Saeki, Takeshi Okamoto, Shingo Higaki, Ikuei Fujii, Chieko Suzuki, Tomomi Hoshida, Toshihiko Matsumoto, Taro Takami, Isao Sakaida, Takahiro Yamasaki

**Affiliations:** 1 Department of Gastroenterology and Hepatology, Yamaguchi University Graduate School of Medicine, Ube 755-8505, Japan; w031ub@yamaguchi-u.ac.jp (E.H.); i020ub@yamaguchi-u.ac.jp (H.F.); g029ub@yamaguchi-u.ac.jp (S.I.); agoto@yamaguchi-u.ac.jp (A.G.); khamabe@yamaguchi-u.ac.jp (K.H.); tisemo1979@yahoo.co.jp (I.S.); tokamoto@yamaguchi-u.ac.jp (T.O.); t-takami@yamaguchi-u.ac.jp (T.T.); sakaida@yamaguchi-u.ac.jp (I.S.); 2 Department of Oncology and Laboratory Medicine, Yamaguchi University Graduate School of Medicine, Ube 755-8505, Japan; hoshidat@yamaguchi-u.ac.jp (T.H.); tm0831@yamaguchi-u.ac.jp (T.M.); t.yama@yamaguchi-u.ac.jp (T.Y.); 3 Faculty of Laboratory Science, Yamaguchi University Graduate School of Medicine, Ube 755-8505, Japan; i002up@yamaguchi-u.ac.jp; 4 Department of Gastroenterology, St. Hill Hospital, Ube 755-8505, Japan; shigaki@sthill-hp.or.jp; 5 Ajisu Kyoritsu Hospital, Yamaguchi 754-1277, Japan; fujii@kyoai.or.jp (I.F.); bells-t2@rc4.so-net.ne.jp (C.S.)

**Keywords:** gastric cancer, droplet digital PCR, liquid biopsy, methylated RUNX3

## Abstract

The main modalities for gastric cancer screening are limited to upper gastrointestinal endoscopy and contrast radiography. The former is invasive, and the latter has high false-negative rates. Thus, alternative diagnostic strategies are required. One solution may be a liquid biopsy. Methylated RUNX3 is a well-known biomarker of gastric cancer but it is very difficult to detect with conventional bisulfite-based methylation assays when only a small amount of serum is available. We developed the combined restriction digital PCR (CORD) assay, a new methylation assay allowing for the counting of as little as one copy of a methylated gene in a small sample of DNA without necessitating DNA bisulfite treatment. We evaluated the sensitivity and specificity of the serum DNA testing of methylated RUNX3 by the CORD assay for the detection of early gastric cancer using 50 patients with early gastric cancer and 61 control individuals. The CORD assay had a sensitivity of 50.0% and a specificity of 80.3% for early gastric cancer. Methylated RUNX3 copies were significantly associated with tumor size, massive submucosal invasion, and lymph-vascular invasion. After the treatment, the median number of methylated RUNX3 copies was significantly decreased. The CORD assay may provide an alternative screening strategy to detect even early-stage gastric cancer.

## 1. Introduction

Gastric cancer was the 5th most common malignancy and the 3rd leading cause of cancer mortality worldwide in 2018, and incidence rates were markedly elevated in eastern Asia, including Mongolia, Japan, and the Republic of Korea [1]. In 2018, the 5-year relative rate of survival in Japan was extremely high (94.9%) for stage I gastric cancer, including early gastric cancer. However, it fell to 68.2% for Stage II, 43.4% for Stage III, and 9.6% for Stage IV cancer [2]. Therefore, early detection is very important to reduce the mortality rate of gastric cancer.

Although tumor markers, carcinoembryonic antigen (CEA), and carbohydrate antigen 19-9 (CA19-9) are useful biomarkers widely utilized for the prediction of tumor recurrence after the curative resection of gastric cancer [3], they are not recommended for the screening of gastric cancer in the National Comprehensive Cancer Network guidelines due to their low sensitivity and specificity [4]. As there remains a lack of useful biomarkers for gastric cancer screening, the main modalities for such screening are limited to upper gastrointestinal endoscopy and the contrast radiography of the stomach [5]. Both modalities have disadvantages: upper gastrointestinal endoscopy is invasive and expensive [5], and the barium contrast radiography of the stomach has low sensitivity in early gastric cancer (about 14%) and a high false-negative rate (about 50%) [6,7]. Thus, it is important to develop highly sensitive and specific assays to detect early gastric cancer that are non-invasive, inexpensive, and easy to perform.

One solution may be a free-circulating DNA test in blood (liquid biopsy). Methylated RUNX 3 is a well-known biomarker of gastric cancer [8]. The methylation of RUNX3 is mediated by *Helicobacter pylori* infection [9], which can lead to inflammation in gastric tissue and may induce atrophy, metaplasia, and dysplasia [10]. About 80% of gastric cancers worldwide are associated with *H. pylori* infection [11]. We have created an analytically sensitive technique, the combined restriction digital PCR (CORD) assay, which is a very sensitive methylation assay allowing for the counting of as little as one copy of a methylated gene in a small sample of DNA without necessitating DNA bisulfite treatment. Its sensitivity for minute quantities of the target methylated gene is more than 100 times higher than that of conventional bisulfite-based methylation assays and thus overcomes the issue of limited sample input [12,13,14].

In the present study, we evaluated the clinical performance of the CORD assay targeting methylated RUNX3 for the detection of early gastric cancer from serum samples.

## 2. Results

### 2.1. CEA, CA19-9, and Serum Anti-H. Pylori Antibody Titer

Increased levels of CEA (> 6 ng/mL) and CA19-9 (> 37 U/mL) were found in 2/50 (4.0%) and 0/50 (0.0%) patients with early gastric cancer, respectively. Increased levels of anti-*H. pylori* antibody titers (>10 U/mL) were found in only 16/50 (32.0%) patients with early gastric cancer because approximately half of the patients (23/50 [46%]) had already received *H. pylori* eradication. Specificity was not available because we did not measure CEA, CA19-9, or anti-*H. pylori* antibody titers in the control group.

### 2.2. Basic Performance Test of the CORD Assay

**Figure 1:**
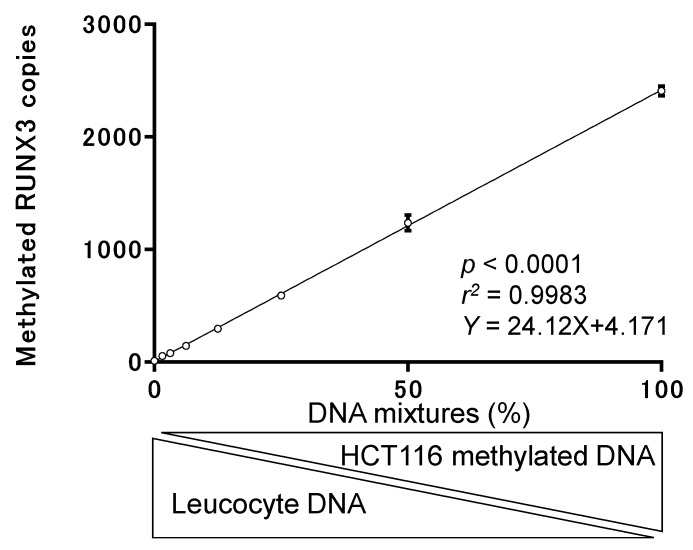
Basic test of combined restriction digital PCR (CORD) assay performance. The *x* axis represents the ratio of methylated HCT116 DNA to leukocyte DNA in the template DNA. The *y* axis represents methylated copy numbers for RUNX3, as determined experimentally.

We tested the basic ability of the CORD assay to detect hypermethylated cancer-derived DNA in comparison with blood-derived DNA by spiking EpiScope methylated HCT116 gDNA (Takara Bio Inc., Japan; control DNA for methylation of RUNX3) into DNA retrieved from leukocyte DNA (control DNA for demethylation of RUNX3) at the following ratios: 100%, 50%, 25%, 12.5%, 6.25%, 3.12%, 1.56%, and 0%. We then measured the levels of RUNX3 methylation in each sample. Figure 1 and Table 1 show that the CORD assay can quantify copy numbers of methylated RUNX3 from 59 pg of control methylated DNA in a background of 3691 pg of control unmethylated DNA. We determined that DNA derived from 40 µL of serum could be used as a template for digital PCR, in which the amount of DNA ranged from 200 to 2750 pg.

### 2.3. Methylated RUNX3 as a Biomarker of Early Gastric Cancer

**Figure 2:**
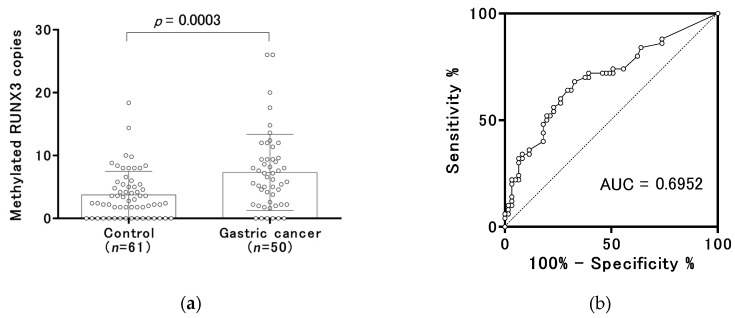
Distribution of methylated RUNX3 copy numbers. (**a**) The distribution of copy numbers of methylated RUNX3 is shown for each group. Open circles indicate the individual samples. The numbers of methylated RUNX3 copies per a DNA amount equivalent to that in 40 µL of serum are shown. The box plots show the median and interquartile range (25th and 75th percentiles). (**b**) The receiver-operating characteristic (ROC) curve analysis of copy numbers of methylated RUNX3 to discriminate between the control group and early gastric cancer group is shown.

**Figure 3:**
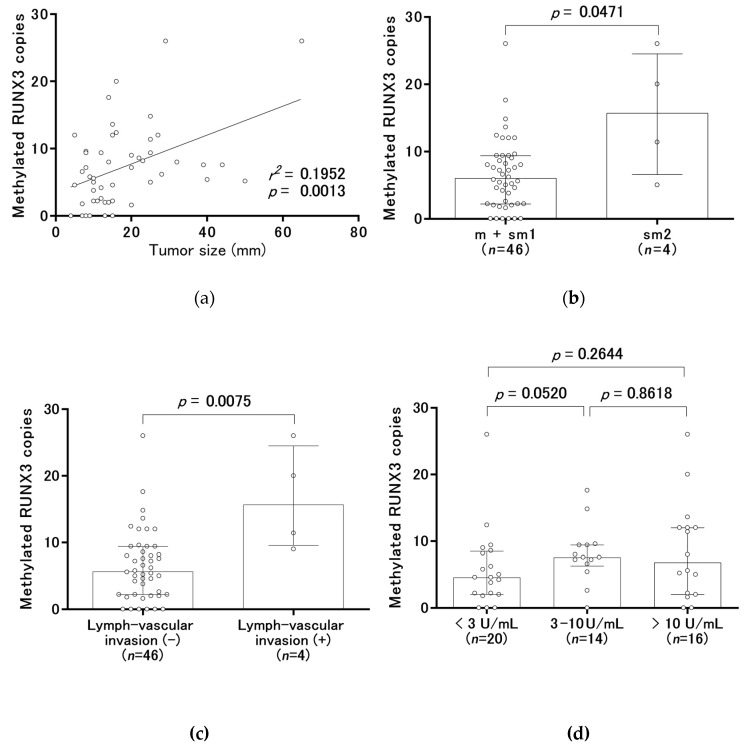

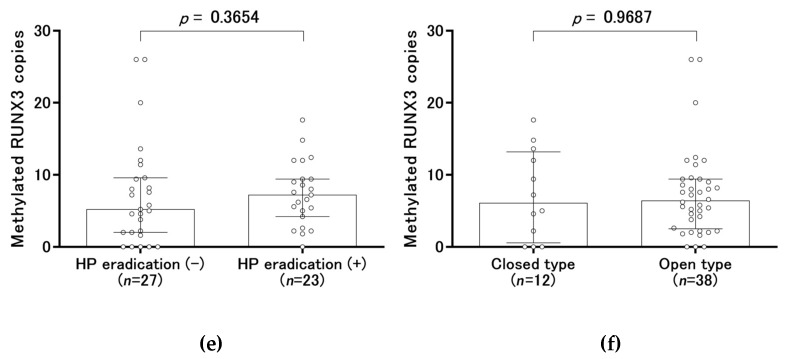
Comparison of tumor characteristics and clinical course with methylated RUNX3 copy numbers. (**a**) The correlation between tumor size and the copy numbers of methylated RUNX3 is shown. In all panels, the numbers of methylated RUNX3 copies per a DNA amount equivalent to that in 40 µL of serum are shown, with open circles indicating the individual samples. (**b**–**f**): the distribution of copy numbers of methylated RUNX3 according to the depth of tumor invasion (**b**), the presence of lymph-vascular invasion (**c**), the titer of *H. pylori* antibody (**d**), the presence of *H. pylori* eradication (**e**), and the severity of endoscopic gastric atrophy (**f**). In panels (**b**–**f**), box plots show the median and interquartile range (25th and 75th percentiles).

The median copy numbers of methylated RUNX3 were 2.8 (range, 0.0 to 18.4) in the control group and 6.4 (range, 0.0 to 26.0) in the gastric cancer group before treatment (Figure 2a). According to receiver-operating characteristic (ROC) curve analysis, the area under the curve (AUC) was 0.6952 (Figure 2b). We set 6.4 copies of methylated RUNX3, which was the median copy number in the gastric cancer group, as a tentative cut-off point. The frequency above the cut-off point was 19.7% (12/61) of the subjects in the control group (specificity of 80.3%) and 50.0% (25/50) in the gastric cancer group. An increase in methylated RUNX3 copy numbers was significantly related to tumor size, massive submucosal invasion (sm2), and lymph-vascular invasion (Figure 3a–c), but not to the *H. pylori* status and the severity of endoscopic gastric atrophy (Figure 3d–f). Binominal logistic regression analysis showed that age, gender, the severity of endoscopic gastric atrophy, and methylated RUNX3 level were independent risk factors for gastric cancer (Table 2).

### 2.4. Changes in Serum-Methylated RUNX3 Copies Before and After Treatment

**Figure 4:**
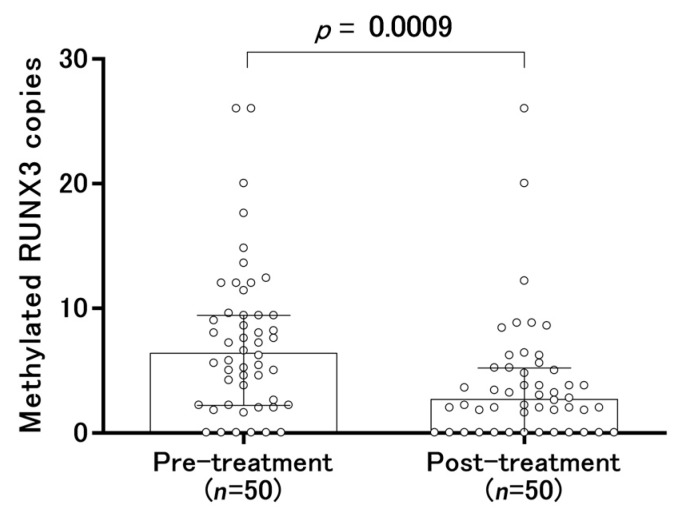
Changes in serum-methylated RUNX3 copies before and after treatment. In the panel, box plots show the median and interquartile range (25th and 75th percentiles).

After endoscopic and/or surgical treatment, the median copy number of methylated RUNX3 was significantly decreased from 6.4 to 2.7 (range, 0.0 to 26.0) (Figure 4). The frequency above the tentative cut-off point (6.4 copies of methylated RUNX3, which was the median copy number before treatment) was 14.0% (7/50) of methylated RUNX3 after the treatment. Although the copy number of methylated RUNX3 decreased in two of the treated patients, it increased in five other patients (Figure 4).

## 3. Discussion

In the present study, serum CEA and CA19-9 showed quite low sensitivity in the detection of early gastric cancer (3.0% and 0.0%, respectively), corresponding to a report by other investigators [4]. Increased anti-*H. pylori* antibody titer (> 10 U/mL) was found in 17/50 (34.0%) patients in the gastric cancer group. However, the prevalence of *H. pylori* infection in Japan is high, and an increased anti-*H. pylori* antibody titer (> 10 U/mL) was found in 604 of 994 (60.8%) subjects in a primary screening [15]. Therefore, the anti-*H. pylori* antibody titer test is inappropriate as a screening test for gastric cancer. In the gastric cancer group, the prevalence rate of increased *H. pylori* antibody titer (> 10 U/mL) was lower as compared to the prevalence of gastric severe atrophy (34.0% vs. 76.0%). This discrepancy may be due to a history of *H. pylori* eradication therapy. Indeed, 23 patients with gastric cancer have a history of *H. pylori* eradication therapy. Furthermore, some of the patients with gastric cancer might have a history of spontaneous elimination of *H. pylori*, which may also be related to the discrepancy.

The bisulfite treatment of DNA is generally performed in conventional methylation assays, but this reaction can introduce various DNA strand breaks, resulting in single-stranded DNA with high fragmentation [16] and a DNA loss of around 90% [17]. After the bisulfite treatment of the DNA, these assays require a minimum of 10 copies of the target gene in the template DNA [18]. Thus, prior to bisulfite treatment, each template DNA would require at least 100 copies of the target gene if an expected 90% of the DNA is lost during the treatment [16]. In contrast, because the serum CORD assay does not require the bisulfite treatment of DNA, the amount of template DNA required is equivalent to that only in 40 µL of serum [13]. The evaluation of the methylation level is performed with droplet digital PCR, which can count even one copy of the target gene [19]. These factors result in an experimental technique that is easier to perform in the CORD assay than in the conventional methylation assays [12,13,14,20,21,22]. The problem of the restriction enzyme-based methylation assay, including the CORD assay, is the possibility of incomplete digestion leading to false positives. To avoid the problem, we added exonuclease I to eliminate single-stranded DNA so that the single-stranded DNA did not undergo PCR amplification. Furthermore, we set the incubation time of the restriction enzymes to 16 h, which is the longest time according to manufacturers’ instructions, to avoid incomplete digestion of DNA, and every time we perfumed the CORD assay, leukocyte DNA was used as an external control for unmethylation of RUNX3 to monitor the completeness of the digestion.

Although circulating methylated RUNX3 measured by the conventional bisulfite-based serum methylation assay in the blood in patients with gastric cancer was reported by other investigators [8,9,23,24], the sensitivity for stage I gastric cancer, including early gastric cancer, is extremely low, ranging from 0.0% to 19.0% [9,24]. The problem with the conventional bisulfite-based serum methylation assay is the lack of adequate sensitivity for minute quantities of circulating tumor DNA. Sakakura et al. used bisulfite-modified DNA and the real-time methylation-specific PCR of RUNX3 consisting of two steps of PCR to increase sensitivity for minute quantities of circulating tumor DNA, which resulted in a sensitivity of 0.0% (0/28) for stage I gastric cancer [24]. Lu et al. used bisulfite-modified DNA and the real-time methylation-specific PCR of RUNX3, which resulted in a sensitivity of 19.0% (4/21) for stage I gastric cancer [9]. In contrast, because the bisulfite treatment of DNA is not required in our CORD assay and it is 100 times more sensitive than the conventional methylation assays, we thought the CORD assay would improve clinical performance. Indeed, the serum CORD assay of methylated RUNX3 resulted in a sensitivity of 50% for the detection of early gastric cancer, showing better performance compared to that in the previous reports (0–19.0%), as mentioned above [9,24].

We found a significant association between increased numbers of methylated RUNX3 copies and lymph-vascular invasion and tumor size in early gastric cancer. Although this finding corresponds with that of another report, that study consisted of stage I-IV cancers, not just that limited to stage I [24]. A very low level of RUNX3 methylation in patients with stage I gastric cancer makes it difficult to compare the methylation level of RUNX3 with lymph-vascular invasion and tumor size [24]. Therefore, to our knowledge, this is the first report to show both an increase in the number of methylated RUNX3 copies with lymph-vascular invasion and tumor size in early gastric cancer and a significant association between an increase in methylated RUNX3 copies and massive submucosal (sm2) invasion. Tumor size, massive submucosal (sm2) invasion, and lymph-vascular invasion are often listed as risk factors for lymph node metastasis in early gastric cancer [25,26,27,28]. As endoscopic resection is applied to the treatment of early gastric cancer without lymph node metastasis [29], the serum circulating methylated RUNX3 copies could be a biomarker to determine whether early gastric cancer is resected endoscopically or surgically.

The present study has some limitations. The sample size is small, and the distribution of age and sex is different between the control and cancer groups. Thus, we consider this to be preliminary data showing the level of methylated RUNX3 circulating in the blood to be a promising biomarker of early gastric cancer. An increase in this level can also be observed in patients with other types of cancer, such as lung, breast, pancreas, colorectum, and liver [8], and even in those with benign disease [24]. Thus, an increase in the number of methylated RUNX3 copies in blood may suggest the presence of some kind of cancer or benign disease, not just that limited to gastric cancer. In the present study, about 20% of the participants in the control group had an increased copy number of methylated RUNX3. The copy number of methylated RUNX3 also remained high in seven patients with gastric cancer after curative treatment. Further medical check-ups and a follow-up survey will be required for the subjects. To clarify the usefulness of the DNA testing of methylated RUNX3 as a universal tumor marker from blood samples, retrospective and prospective cohort studies comprising various types of cancer will be required.

The use of the CORD assay is especially advantageous when using biobank resources to perform retrospective studies because the amounts in commercially available blood samples from biobanks are small, usually less than 1 mL [30]. Up to 4 mL of plasma or serum can be required for the conventional methylation assays of laboratory testing [21,22,31], whereas for a single test, the CORD assay only requires a DNA amount equivalent to that in 40 µL of serum. Thus, the measurement of the methylation levels of various genes in archived blood samples from a biobank and confirmation of the reproducibility of data are much easier with the CORD assay.

In conclusion, the RUNX3 methylation analysis by serum CORD assay showed moderate sensitivity and moderately high specificity for the detection of early gastric cancer. Because this study suggests that the serum DNA testing of methylated RUNX3 by the CORD assay may be useful to detect individuals with early gastric cancer, confirmatory studies using independent data sets with larger sample sizes will be needed to support our findings.

## 4. Materials and Methods

### 4.1. Materials

Serum was prospectively collected in advance of upper gastrointestinal endoscopy between November 25, 2016 and February 29, 2020 in Yamaguchi University Hospital, St. Hill Hospital, or Ajisu Kyoritsu Hospital, and was stored at −80 °C until DNA extraction. To avoid the artificial contamination of the blood by cancer cells, blood collection was performed at least 3 weeks after the biopsy if the biopsy had been performed. We also collected serum samples more than 2 months after the endoscopic or surgical treatment. The cancer subjects comprised 50 patients with early gastric cancer diagnosed by endoscopic and/or surgical resection who were curatively treated and had no recurrence of gastric cancer or occurrence of cancers from other organs. We recruited asymptomatic persons who were scheduled to undergo upper gastrointestinal endoscopy for medical check-up as potentially eligible control subjects. The control subjects comprised 61 healthy volunteers with no previous history of any type of cancer and who were without gastric cancer as determined by upper gastrointestinal endoscopy. All subjects were Japanese. The depth of tumor invasion was classified into mucosal (m), submucosal microinvasion (sm1) (< 500 μm penetration into submucosa), and massive submucosal invasion (sm2) (≥ 500 μm) [32]. Well differentiated or moderately differentiated tubular adenocarcinoma and papillary adenocarcinoma were classified as differentiated carcinoma, whereas poorly differentiated tubular adenocarcinoma and signet-ring cell carcinoma were classified as undifferentiated carcinoma. Lesions containing both differentiated and undifferentiated carcinoma were classified based on whichever microscopic type was dominant [32]. The severity of gastric atrophy was investigated by using the Kimura-Takemoto classification [33]. The staging was classified according to the International Union Against Cancer (UICC) [34].

In addition to the explanation in the consent form, we verbally explained the purpose of this study and the complications and risks of upper gastrointestinal endoscopy in face-to-face interviews with the subjects. All healthy volunteers participated spontaneously, clearly understood the risks of upper gastrointestinal endoscopy, and signed the written informed consent form. Table 3 shows the patients’ clinicopathologic characteristics.

The institutional review boards of Yamaguchi University Graduate School of Medicine, St. Hill Hospital, and Ajisu Kyoritsu Hospital approved this study protocol (approval no. H28-124). Written informed consent was obtained from all patients and healthy volunteers.

### 4.2. Carcinoembryonic Antigen

CEA was measured in the 50 early gastric cancer patients with a “TOSOH” II CEA commercial immunoassay kit and an AIA-2000 automatic immunoassay analyzer (Tosoh Corporation, Tokyo, Japan) in the laboratory of Yamaguchi University Hospital. We set the cutoff value for the serum CEA level at 6 ng/mL in accordance with the manufacturer’s instructions.

### 4.3. Serum Carbohydrate Antigen 19-9

CA19-9 was measured in the 50 early gastric cancer patients with an “ARCHITECT CA19-9XR” CA19-9 commercial immunoassay kit and an ARCHITECT i2000 automatic immunoassay analyzer (Abbot Corporation, Tokyo, Japan) in the laboratory of Yamaguchi University Hospital. We set the cutoff value for the serum CA19-9 level at 37 U/mL in accordance with the manufacturer’s instructions.

### 4.4. Serum Anti-H. Pylori Antibody Titer

The measurement of serum anti-*H. pylori* antibody in the 50 patients with early gastric cancer was conducted by LSI Medience Corporation, a clinical laboratory testing service in Tokyo, Japan. A commercial kit (LZ test “Eiken” *H. pylori* antibody; Eiken Kagaku, Tokyo, Japan) and a BM9130 automatic clinical chemistry analyzer (JEOL, Tokyo, Japan) were used to measure antibodies against *H. pylori*. An antibody titer cutoff point of >10 U/mL was used to evaluate *H. pylori* infection [11,35]. We divided patients into three groups according to their serum *H. pylori* antibody titers: < 3 U/mL, 3–10 U/mL, and > 10 U/mL [36].

### 4.5. Preparation of Samples and DNA Extraction

DNA from peripheral blood leukocytes was used as a control for unmethylated RUNX3, and EpiScope methylated HCT116 gDNA (Takara Bio Inc.) was used as a control for hypermethylated RUNX3. After the serum samples were thawed from −80 °C, DNA was extracted from 0.4 mL of each sample with a MagNA Pure Compact Nucleic Acid Isolation Kit I (Roche, Tokyo, Japan) in accordance with the manufacturer’s instructions. The DNA was eluted in 50 µL of elution buffer, and Qubit 2.0 fluorometers (Thermo Fisher Scientific, Yokohama, Japan) were used to quantify the double-stranded DNA.

### 4.6. CORD Assay

The CORD assay consists of two steps: the treatment of DNA with multiple methylation-sensitive restriction enzymes and then multiplex digital PCR as previously described [12,13]. The first step of the enzyme treatment consisted of the digestion of 10 µL of eluted DNA (a DNA amount equivalent to that in 80 μL serum) for 16 h at 37 °C by adding 1 µL of GeneAmp 10 × PCR Buffer II, 1 µL of 25 mmol/L MgCl_2_, 10 U each of Hha I and Hpa II, and 20 U exonuclease I (Exo I) (all, Thermo Fisher Scientific). Exo I was used to eliminate single-stranded DNA that escaped digestion by the restriction enzymes and to ensure that the undigested fraction did not undergo PCR amplification [37]. The second step consisted of the additional digestion of DNA being performed for 16 h at 60 °C using 10 U of BstUI (New England Biolabs Ltd., Hitchin, UK). After the completion of restriction, the mixture underwent heating for 10 min at 98 °C. The RUNX3 had a recognition site (CCGG) of methylation-sensitive enzyme Hpa II, and when that site was methylated, the target DNA would escape digestion by the enzyme and would thus be amplified by PCR. We performed droplet digital PCR to count the absolute copy numbers of methylated RUNX3. The PCR reaction solution was prepared by combining 8 µL of enzyme-treated DNA (a DNA amount equivalent to that in 40 µL of serum), 1 × ddPCR Supermix for Probes (BioRad, Tokyo, Japan), 0.25 µmol/L of each primer, and 0.125 µmol/L of the RUNX3 probe in a total volume of 20 µL. The primer and probe set sequences for RUNX3 were as follows: forward primer, 5’-TATGCGTATTCCCGTAGACCC-3’; reverse primer, 5’-GCTGTTCTCGCCCATCTTG-3’; and probe, 5’-FAM-TCCCCGGCCTTCCCCTGCGG-TMARA-3’. The PCR amplicon length is 100 bp from 25,256,261-25,256,360 of chromosome 1 (human assembly GRCh37/hg19). We re-designed the RUNX3 primers and probe on the basis of a previous report in which differentially methylated RUNX3 in gastric cancer is designated [8]. After droplet generation was completed by an automated droplet generator (BioRad), PCR was performed using the following cycling conditions: preheating at 95 °C for 10 min, 40 cycles of denaturation at 94 °C for 30 s, annealing at 56 °C for 60 s, and final heating at 98 °C for 10 min. Following amplification, the PCR plate was placed in a QX100 droplet reader (BioRad) and fluorescence amplitude data were analyzed by QuantaSoft software (BioRad).

### 4.7. Statistical Analyses

Variables were compared with the Mann–Whitney U test, paired *t*-test, Fisher’s exact test, and ROC curve analysis, and linear regression analyses were also performed. A *p* value < 0.05 was considered to indicate statistical significance. GraphPad Prism Ver. 6 software and GraphPad InStat Ver. 3 were used for the statistical analyses (GraphPad Software, La Jolla, CA). Binominal logistic regression analysis was performed to identify independent risk factors for gastric cancer using BellCurve for Excel (Social Survey Research Information Co., Ltd., Tokyo, Japan).

## 5. Conclusions

RUNX3 methylation analysis by serum CORD assay showed moderate sensitivity and moderately high specificity for the detection of early gastric cancer. Because this study suggests that the serum DNA testing of methylated RUNX3 by the CORD assay may be useful in detecting gastric cancer, the CORD assay may provide an alternative screening strategy to detect even early-stage gastric cancer.

## Figures and Tables

**Table 1:** Summary of basic test of CORD assay performance.

Factors	Mixtures (%)							
	0	1.56	3.13	6.25	12.5	25	50	100
Amount of template DNAs (pg)								
HCT116 methylated DNA	0	59	117	234	469	938	1875	3750
Leukocyte DNA	3750	3691	3633	3516	3281	2813	1875	0
Measured methylated RUNX3								
Mean copy numbers	11	54	78	144	296	592	1237	2408
SD	3.4	10.6	9.8	13.4	27.5	28.9	69.7	43.8

**Table 2:** Risk factors for gastric cancer.

Factors	Univariate Analysis		Multivariate Analysis	
	OR (95% CI)	*p*-Value	OR (95% CI)	*p*-Value
Age in years	1.11 (1.06–1.16)	< 0.001	1.09 (1.03–1.15)	0.0048
Gender				
Male	4.13 (1.71–9.95)	0.0013	7.47 (2.05–27.23)	0.0023
Female	Reference			
Gastric atrophy				
Open type	18.30 (7.00–47.80)	<0.001	9.50 (2.97–30.35)	< 0.001
Closed type	Reference			
Methylated RUNX3 level				
>6.4 copies	4.08 (1.76–9.46)	0.0011	4.43 (1.38–14.28)	0.0126
≤6.4 copies	Reference			

OR: odds ratio, CI; confidence interval.

**Table 3:** Clinicopathologic characteristics of the subjects.

Parameters	Category	Gastric Cancer (*n* = 50)	Control (*n* = 61)	*p*-Value
Age in years	Median (range)	72.2 (34–90)	58 (39–86)	<0.0001
Sex	Male	41	32	0.0013
	Female	9	29	
Methylated RUNX3	Median (range)	6.4 (0.0–26.0)	2.8 (0.0–18.4)	0.0003
Gastric atrophy	Closed type	12	52	<0.0001
	Open type	38	9	
Tumor size (mm)	Median (range)	14.5 (4.0–65.0)	NA	NA
Depth of tumor invasion	m	42	NA	NA
	sm1	4		
	sm2	4		
Tumor differentiation	Differentiated	46	NA	NA
	Undifferentiated	4		
Lymph-vascular invasion	Present	4	NA	NA
	Absent	46		
History of *H. pylori* eradication	Present	23	NA	NA
	Absent	27		
Anti-*H. pylori* antibody titer	>10 U/mL	16	NA	NA
	3–10 U/mL	14		
	<3 U/mL	20		
CEA	>6.0 ng/mL	2	NA	NA
	≤6.0 ng/mL	48		
CA19-9	>37.0 U/mL	0	NA	NA
	≤37.0 U/mL	50		

NA, not available. m, mucosa. sm, submucosa. *H. pylori*, *Helicobacter pylori*.
